# Effects of a Theta/Sensorimotor Rhythm Neurofeedback Training Protocol on Measures of Impulsivity, Drug Craving, and Substance Abuse in Forensic Psychiatric Patients With Substance Abuse: Randomized Controlled Trial

**DOI:** 10.2196/10845

**Published:** 2018-12-11

**Authors:** Sandra Fielenbach, Franc CL Donkers, Marinus Spreen, Stefan Bogaerts

**Affiliations:** 1 Research Department Forensic Psychiatric Centre Dr S van Mesdag Groningen Netherlands; 2 Department of Developmental Psychology Tilburg University Tilburg Netherlands; 3 Department of Cognitive Neuroscience Maastricht University Maastricht Netherlands; 4 Fivoor Science and Treatment Innovation Poortugaal Netherlands

**Keywords:** neurofeedback, impulsivity, substance use disorder, offenders, drug craving

## Abstract

**Background:**

Forensic psychiatric patients are often diagnosed with psychiatric disorders characterized by high levels of impulsivity as well as comorbid substance use disorders (SUD). The combination of psychiatric disorders and SUD increases the risk of future violence. Chronic substance abuse can lead to a structural state of disinhibition, resulting in more drug taking and eventually loss of control over drug intake. When treating SUD, it is crucial to address high levels of impulsivity and lack of inhibitory control.

**Objective:**

This study set out to investigate the effects of a theta/sensorimotor rhythm (SMR) neurofeedback training protocol on levels of impulsivity, levels of drug craving, and actual drug intake in a population of forensic psychiatric patients with a diagnosis of SUD.

**Methods:**

A total of 21 participants received 20 sessions of theta/SMR neurofeedback training in combination with treatment-as-usual (TAU). Results of the intervention were compared with results from 21 participants who received TAU only.

**Results:**

SMR magnitude showed a significant (*P*=.02) increase post training for patients in the neurofeedback training group, whereas theta magnitude did not change (*P*=.71). Levels of drug craving as well as scores on the *motor* subscale of the Barratt Impulsivity Scale-11 decreased equally for patients in the neurofeedback training group and the TAU group. Other measures of impulsivity as well as drug intake did not change posttreatment (*P*>.05). Therefore, neurofeedback+TAU was not more effective than TAU only.

**Conclusions:**

This study demonstrated evidence that forensic psychiatric patients are able to increase SMR magnitude over the course of neurofeedback training. However, at the group level, the increase in SMR activity was not related to any of the included impulsivity or drug craving measures. Further research should focus on which patients will be able to benefit from neurofeedback training at an early stage of the employed training sessions.

**Trial Registration:**

Dutch National Trial Register: NTR5386; http://www.trialregister.nl/trialreg/admin/rctview.asp?TC=5386 (Archived by WebCite at http://www.webcitation.org/6nXLQuoLl).

## Introduction

### Background

Forensic psychiatric patients are often times diagnosed with disorders characterized by high levels of impulsivity. Schizophrenia, attention-deficit hyperactivity disorder (ADHD), and cluster B personality disorders are the most common disorders in forensic psychiatric patients [1,2]. Substance use disorder (SUD) is a common comorbidity, occurring in about 55% to 70% of all patients [2,3].

Individuals abusing alcohol, stimulants, and opioids tend to have higher levels of impulsivity as compared with nonabusing controls [4]. Furthermore, impulsivity is a risk factor for the development and maintenance of SUD [5,6]. Chronic substance abuse can cause a structural state of disinhibition over time, leading to permanent excessive abuse of substances [7-9]. This state is not limited to the acute stages of substance dependency but is also present in patients after stopping regular drug intake [10]. Elevated levels of impulsivity are also associated with more severe symptoms of SUD, which eventually lead to higher levels of drug craving [11]. Once patients receive substance abuse treatment, high levels of impulsivity can increase chances of early relapse and premature termination of treatment [6,10]. For forensic psychiatric patients, substance use is highly associated with the use of violence, regardless of the type of drug used [12-14].

Important in the treatment of forensic patients with SUD is to determine levels of impulsivity and lack of inhibitory control. In accordance with this, common psychotherapeutic approaches for SUD involve the adaptation of strategies that promote conscious decision making, attention to action, and control over behavior [9]. Despite that, relapse rates following remission of treated SUD individuals are as high as 60% [15], stressing the need for additional interventions.

### Neurofeedback Treatment for Impulsivity and Substance Use Disorder

In the last two decades, electroencephalographic (EEG) neurofeedback training has shown promising results in reducing high levels of impulsivity in patients suffering from ADHD [16-18]. Neurofeedback training uses real-time EEG measurements and displays this information back to the patient [19]. EEG neurofeedback training works by enhancing or inhibiting brain frequencies that have shown to underlie abnormal psychological states [20]. A frequently used neurofeedback protocol to train impulse control is the so-called theta (3.5-7.5 Hz)/sensorimotor rhythm (SMR, 12-15 Hz) protocol [17,21,22]. In this protocol, the magnitude of the SMR frequency is enhanced, whereas the magnitude of the theta frequency is inhibited.

However, the effectiveness of a theta/SMR neurofeedback protocol on levels of impulsivity and also on symptoms of SUD such as levels of craving and actual drug use in forensic psychiatric patients is unclear. Only a few studies have investigated the effects of neurofeedback training in forensic psychiatric patients [23-26]. Therefore, investigating effectiveness of neurofeedback training can add value to treatment models that are currently used for this group, such as classical psychotherapy and pharmacological treatment.

### Objectives

The primary objective of this study was to examine to what extent a theta/SMR neurofeedback training results in the reduction of impulsivity, drug craving, and actual drug use in a population of forensic psychiatric patients with a diagnosis of SUD. Patients were allocated to either a neurofeedback-training group where they received 20 sessions of a theta/SMR neurofeedback training protocol in addition to treatment-as-usual (TAU), or a TAU-only group. For both groups, levels of impulsivity, as measured with the Barratt Impulsivity Scale-11 (BIS-11) [27], and a cued Go/No-Go reaction time task [28] were assessed. Levels of drug craving were measured with a modified version of the Desire for Alcohol Questionnaire (DAQ-SF) [29]. Actual drug intake was assessed with urine or breathalyzer drug testing. Results on primary outcome measures were compared between groups We hypothesized that patients receiving neurofeedback training+TAU would show reduced levels of impulsivity post training as well as reduced levels of drug craving and drug use in comparison with patients receiving TAU only.

## Methods

### Study Design and Participants

This study reports the results from a randomized controlled trial (RCT) as described in the study by Fielenbach et al [30]. The results of the n-of-1 clinical case series will be reported elsewhere.

The study took place in a maximum security inpatient forensic psychiatric center (FPC) in Groningen, the Netherlands. All patients in this treatment facility are male criminal offenders who were held only partially responsible for the crime they committed due to severe mental illness, according to Dutch jurisdiction [31]. Inclusion criteria were the presence of at least one diagnosis of SUD according to the Diagnostic and Statistical Manual of Mental Disorders, Fourth Edition, Text Review (DSM-IV-TR [32]), positive drug testing in the past 24 months before inclusion, and sufficient knowledge of the Dutch language to understand training instructions. All patients had at least one comorbid axis I and/or II diagnosis. Exclusion criteria were a state of acute psychosis, in which patients experienced severe delusions and/or hallucinations and could possibly become a threat to themselves or others (a diagnosis of schizophrenia as well as disorders in the schizophrenia spectrum [eg, schizoaffective disorder] were not considered exclusion criteria). A comorbid diagnosis of epilepsy and visual and/or auditory impairments, which would hamper patients’ ability to follow instructions and adhere to the neurofeedback training, were also exclusion criteria. Medication intake was not restricted. Patients were allowed to continue the use of medication over the course of the study. Treatment supervisors were informed that, during the course of the study, prescribed medication should preferably remain stable and that a change in type as well as dosage of medication should not be made during the course of the study unless absolutely necessary. Treatment supervisors were asked to inform researchers in case a change in type or dosage of medication did occur.

This study was conducted according to the principles of the Declaration of Helsinki (version 59, Seoul, October 2008) and in accordance with the Medical Research Involving Human Subjects Act. It is ethically approved by the medical ethical council of Brabant, the Netherlands (study number NL46390.008.13).

### Procedure

In this study, a pre-post test design was used. A power analysis calculation for the RCT, using G*Power 3 based on a 1-tailed alpha value of .05, a power value of 0.80, and an effect size (*f*) of 0.80, yielded a recommended sample size of 21 participants each in the control and intervention conditions.

**Figure 1:**
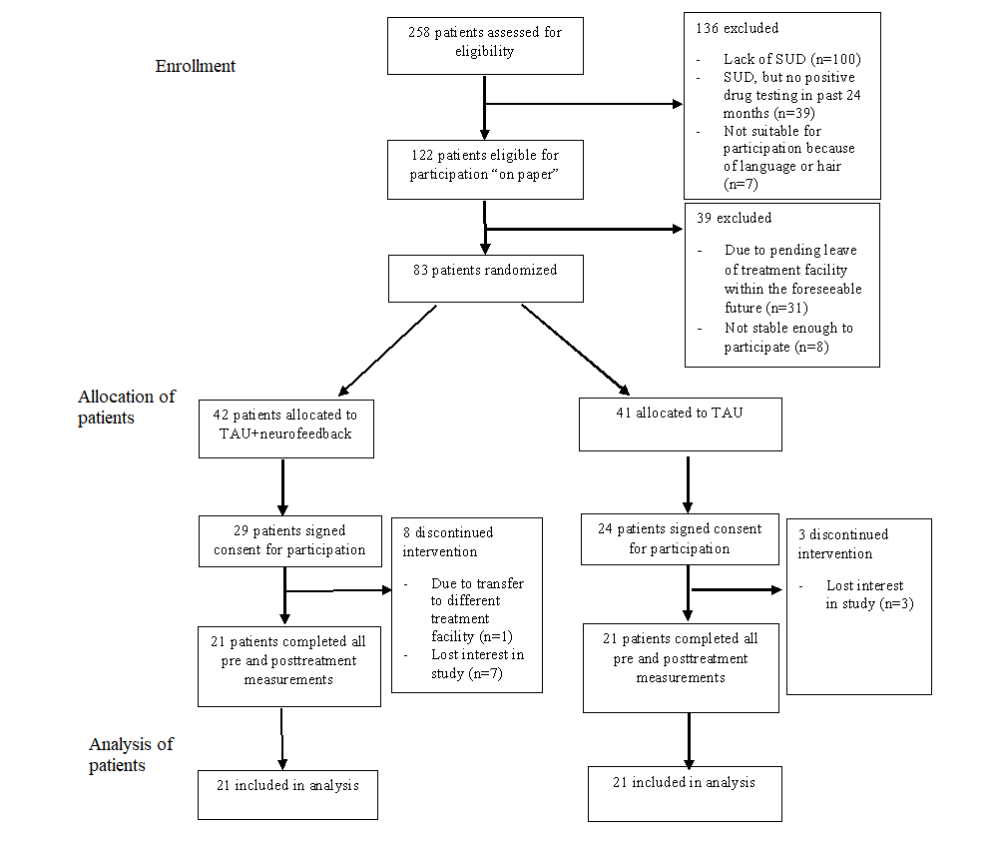
Consolidated Standards of Reporting Trials (CONSORT) flow diagram for individual randomized controlled trials of nonpharmacologic treatments. Patients who had hair that was unsuitable for conducting electroencephalographic measurements or placement of neurofeedback electrodes, such as dreadlocks, were excluded. For analysis of drug testing, data from 19 patients were used. SUD: substance use disorders; TAU: treatment-as-usual.

Out of all participants that met the requirements, a random sample was drawn and randomly assigned to 1 of the 2 study conditions (neurofeedback training+TAU or TAU only). Patients were approached through treatment supervisors for participation and informed about the general outline of the study. If they expressed interest in participating in the study, they were approached by 1 of the researchers to explain the study design and randomization procedure. All patients signed the informed consent. Randomization was done by a random number generator (see Figure 1 for an overview of patient flow through the study).

Participants in both conditions underwent pretreatment measurements (T0), consisting of the measurements described below. Participants in the control group received TAU only. TAU was different for every patient, as treatment modalities are based on individual diagnosis and problematic behavior of the patient as well as the cognitive ability to undergo different treatment modalities. Examples of treatment modalities were nonverbal therapy forms (eg, psychomotor therapy and musical therapy) and cognitive-behavioral group therapy. After 10 weeks, participants in the control group underwent posttreatment measurements (T1), identical to pretreatment measurements.

Once pretreatment measures were completed, participants in the intervention group started the neurofeedback training. They received 20 neurofeedback training sessions, scheduled 2 times a week for 10 weeks. Neurofeedback training was conducted by a certified neurofeedback therapist. The study was not blinded, as it was clear to patients as well as the neurofeedback therapist which patients received the neurofeedback training.

Participants received a small financial compensation comparable with minimum wage in the treatment facility for participation.

### Neurofeedback Training Protocol

For neurofeedback training, electrode Cz was used as the feedback electrode recorded with Ag/AgCl electrodes against a right ear mastoid reference and a FPz ground electrode. Neurofeedback was applied as implemented in the Brainmarker software engine (BrainMarker Device, Brainmarker BV Gulpen). A theta/SMR protocol was used, in which SMR (12-15 Hz) should be enhanced and theta (3.5-7.5 Hz) should be inhibited. If excess high beta (20-32 Hz) or delta (0.5-3.5 Hz) was detected, these frequency bands were inhibited as well, with a maximum of 3 frequency bands being trained in each session. Patients were shown simple video games and instructed to find the most successful strategy to make the main character of the video game move. A movie-based neurofeedback paradigm was given as well, where patients had to stop black curtains from appearing over the computer monitor. The software provided visual positive feedback for increasing SMR magnitude and decreasing theta magnitude. Each round (or trial) of video games lasted 60 seconds, with short breaks in between rounds (trials). Movie-based feedback lasted 90 seconds at a time. The switch between video- and movie-based feedback was done to make neurofeedback more fun and less tiring, as choice of video games provided within the software was limited, as well as very simplistic. For each patient, about 10 rounds of video game-based feedback were employed. As for movie-based feedback, about 10 to 15 rounds were employed. Neurofeedback training lasted for approximately 45 min, including preparation and cleanup.

**Figure 2:**
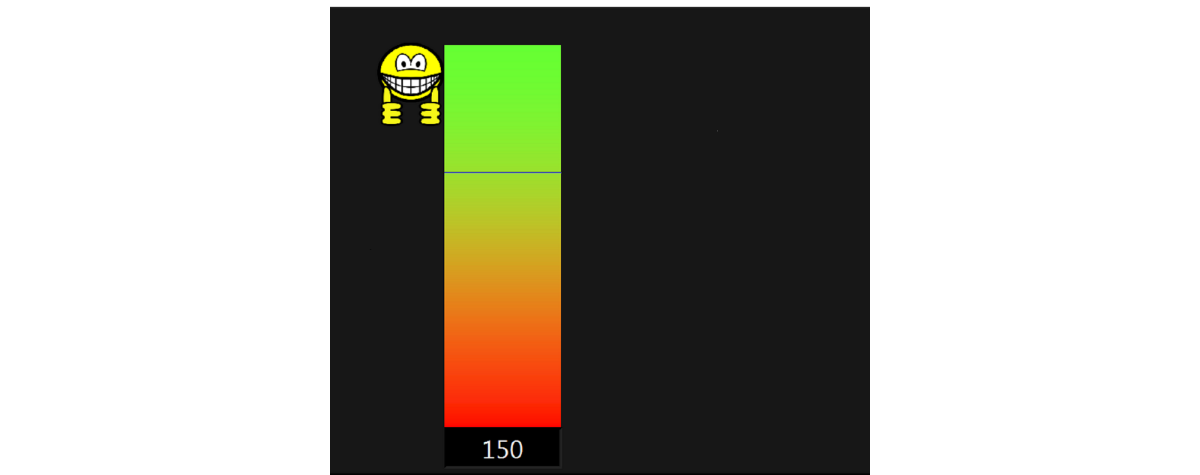
Impression of one of the games used for neurofeedback training using one frequency band. Participants had to try to exceed the bar above the threshold, after which an encouraging smiley popped up on the screen, giving immediate positive reinforcement.

Feedback thresholds were adjusted manually whenever participants were able to increase or decrease the desired frequency bands for 80% of the time. Participants were verbally encouraged to try to move the main character in the video game as much as possible as well as to keep the monitor free from the curtains during video-based feedback and not just stare at the screen (see Figure 2 for an impression of one of the neurofeedback games). After all training sessions were completed, participants underwent posttreatment measurements (T1).

### Measures

#### Electroencephalography

A 5 min 21-channel EEG resting-state measurement with eyes closed was conducted with Nexus-32 hardware and Biotrace software (MindMedia BV). EEG measurements were collected from 19 standard 10/20 positions [33] and the right and left mastoids with a sampling rate of 512 samples per second. The right mastoid served as the online reference. Flat type electrodes were placed above and below the left eye and at the outer canthi of each eye to correct for vertical and horizontal eye movements. EEG magnitude across delta (0.5-3.5 Hz), theta (3.5-7.5 Hz), alpha (7.5-12 Hz), beta (12-20 Hz), SMR (12-15 Hz), high beta (20-32 Hz), and gamma (32-49 Hz) frequency bands was assessed. Magnitude changes in delta, theta, SMR, and high beta frequency were used for analysis. For analysis, custom-made Matlab scripts (version R2012b) were used. First, data from the resting-state measurements were imported into EEGLAB, bandpass filtered between 1-40 Hz, and inspected for gross movement artifacts that were then manually removed. Subsequently, epochs of 4 seconds length were created. Epochs containing amplitudes exceeding ±100 μV at any scalp electrode and/or epochs containing abnormally distributed data (ie, joint probability or kurtosis >5 SD from expected mean values) were rejected. From the remaining epochs, the first 40 were transformed into FieldTrip format (version 20160620). Power values for electrode Cz were computed using a fast Fourier analysis with a Hanning taper as implemented in FieldTrip.

Mean power values for delta, theta, SMR, and high beta frequency bands were calculated and transferred to SPSS for statistical analysis.

#### Barratt Impulsivity Scale-11

The Dutch version of the BIS-11 [27] is a 30-item self-report questionnaire that assesses common impulsive behaviors and preferences across 3 second-order factors: motor, attentional, and nonplanning. An example of a BIS-11 item is “I do things without thinking,” and items are scored across a 4-point Likert scale ranging from “rarely/never” to “almost always/always.” The BIS-11 is an internally consistent measure of impulsivity among inmate populations (Cronbach alpha=. 80) [34].

#### Cued Go/No-Go Task

The cued Go/No-Go task is a continuous performance test designed to measure response inhibition [28]. The task was programmed in E-Prime (version 2.0.10.353). Participants are instructed to respond as quickly as possible to a green square appearing on a screen (“Go target”) but to inhibit responses to a blue square (“No-Go target”). The test consists of 250 targets with equal numbers of Go and No-Go targets. Each target is preceded by either a Go or a No-Go cue, indicating the likelihood of a Go or No-Go target to appear. The likelihood of a correct target after a cue is manipulated with a 80/20 ratio, with 80% being a correct cue and 20% being an incorrect cue. Cues are presented with 4 fixed stimulus onset asynchronies (100, 200, 300, and 400 milliseconds). The program displays feedback about the accuracy of the response back to the participant as well as the time (in milliseconds) it took for the patient to respond to the target. Outcome measure is the number of commission errors, reflecting the failure to inhibit a prepotent response to a No-Go square. Number of commission errors in a cued Go/No-Go task is a valid measure of impulse control in a substance abusing population [28].

#### Modified Version of the Desire for Alcohol Questionnaire Short Form

The short form of the DAQ-SF is a self-report questionnaire that measures levels of craving for alcohol among 14 items scored on a 7-point Likert scale (ranging from 1=strongly disagree to 7=strongly agree). The DAQ-SF has been shown to be a reliable measure to assess craving in a substance-dependent population (Cronbach alpha=.70) [35]. For the purpose of this study, items from the Dutch version of the questionnaire [29] were modified to measure desire for drugs in general, as opposed to being restricted to measure desire for alcohol only. An example of a modified item is “All my tension would completely disappear if I drank now” into “All my tension would completely disappear if I used drugs now.” The modification was made due to the fact that alcohol use is very rare in an inpatient setting, whereas use of other drugs (eg, cannabis or cocaine) is more common.

#### Drug Use

Scores on urine or breathalyzer drug testing were collected for each participant. Drug testing was performed regularly for each patient as part of treatment facility policy. Drug use was operationalized as any positive scoring for use of illegal substances. Illegal substances included all known drugs as well as alcohol and nonprescribed medication used as recreational drug consumption (eg, inhaled methylphenidate). According to treatment facility policy, refusal to undergo drug testing was scored as positive drug testing. To score substance abuse, the item “substance abuse” on the risk assessment scale Historische, Klinische, Toekomst-Revised (Historical, Clinical, Future-Revised) was used [36,37]. This item was scored on a 5-point scale, indicating number of positive drug testing as well as willingness to undergo drug testing. Scores ranged from 0 (no drug use whatsoever) to 4 (the patient tested positive at least twice and also refused to undergo drug testing).

### Data and Statistical Analysis

All participants who completed pre- and posttreatment measures were included in the statistical analysis (n=42). For analysis of drug testing, weekly reports of 2 of the patients from the control group were not available; therefore, the analysis of drug testing consists of data from 40 patients. All data were analyzed with SPSS version 25 (IBM Corp).

Due to violations of statistical assumptions concerning normality and homoscedasticity of almost all dependent variables (BIS motor, BIS attentional, BIS total score, DAQ-SF, delta magnitude, theta magnitude, SMR magnitude, and Cued Go/No-Go commission errors), nonparametric tests were employed. To test for differences between treatment conditions pretreatment, Mann-Whitney U tests were performed for pretreatment scores on BIS-11 total score, BIS-11 subscales “motor,” “nonplanning,” and “attentional” as well as scores on DAQ-SF, number of commission errors, drug testing, and mean theta and SMR magnitude. A Wilcoxon signed-rank test was performed to assess changes within groups between pre- and posttreatment for all dependent variables.

A repeated-measures analysis of variance with time as within-subject variable and treatment condition as a between-subject variable was performed. To assess significance, a within-groups effect size was used (eta squared [η^2^]). Cutoff scores were used according to Cohen’s rules to assess whether effect size were small (η^2^=0.02), medium (η^2^=0.13), or large (η^2^=0.26) [38].

Pearson correlations were performed to test for relations between changes in delta, SMR, high beta, and theta frequency magnitude pretreatment versus posttreatment and all behavioral measures. Only results significant at the .05 level will be reported.

## Results

### Patient Flow

Of those assessed (N=258), 52.71% of patients (136/258) were excluded due to not fitting the inclusion criteria. Moreover, 47.3 % (122/258) of patients were eligible for participation. Those eligible were randomly assigned to either the neurofeedback training+TAU group (n=42) or TAU only group (n=41). Figure 1 summarizes the flow of participants throughout the study.

In total, 42 patients completed all posttreatment measurements, of which 21 patients participated in the TAU only group and 21 patients in the neurofeedback training+TAU group. None of the patients in the neurofeedback training+TAU group were able to complete training within the scheduled 10 weeks. This was due to holidays and planning issues and also because some patients were mentally unable to complete a training session or caused aggressive incidents, which resulted in temporary separation/placement on a specialized crisis unit. It sometimes also happened that patients were unmotivated to attend a training session. Participation in the study, therefore, lasted for an average of 14.1 (SD 5.32) weeks per patient.

When pretreatment measurements were assessed, mean number of months in treatment was 93.6 months (SD 67.18). The large SD was caused by 5 patients who had already been hospitalized for more than 200 months in the treatment facility. Participants did not differ with regard to mean age between the neurofeedback training+TAU group (mean 38.00, SD 9.18) and TAU only group (mean 38.57, SD 8.41; *t*_40_=−0.22, *P*=.63) or mean number of axis I and II DSM-IV-TR diagnoses (neurofeedback training group mean 4.52, SD 1.47; TAU only group mean 4.57, SD 1.63, *t*_40_=−0.09, *P*=.38) or month in treatment before inclusion (neurofeedback training group mean 91.90, SD 61.70; TAU only group mean 95.30, SD 73.76, *t*_40_=−0.16, *P*=.87; see Tables 1-3 for sample characteristics).

### Baseline Differences Between Groups

A Mann-Whitney *U* test indicated that scores on SMR magnitude on pretreatment measurements were significantly higher for patients in the neurofeedback training group (median=1) than for patients in the TAU group (median=.58), *U*=131.00, *P*=.02, *r*=.35.

### Differences Within Groups

Only the neurofeedback training group showed significant effects between pre- and posttreatment scores. Within-groups differences on the BIS-11 subscale *motor* (pretreatment: median=23) showed a significant decrease posttreatment for patients in the neurofeedback training group (median=21, *Z*=2.076, *P*=.04, *r*=.45), as well as a significant decrease in craving scores posttreatment (median=34) as measured with the DAQ-SF (*Z*=2.091, *P*=.04, *r*=.46). SMR mean amplitude also significantly increased from pretreatment (median=1) to posttreatment (median=1.22; *Z*=2.068, *P*=.04, *r*=.45).

### Outcome Measures

The main outcome measures are presented in Table 4. On the primary outcome measures, results on the *motor* subscale of the BIS-11 showed a significant effect for Time (*F*_1,40_=5.61, *P*=.02) but not for Time x Group (*F*_1,40_=1.28, *P*=.28). For the drug craving measure DAQ-SF, there was a significant effect for Time (*F*_1,40_=6.23, *P*=.02) but not for Time x Group (*F*_1,40_=9.2, *P*=.34). There was a significant Time x Group effect for mean SMR magnitude (*F*_1,40_=5.47, *P*=.02), indicating an increase for mean SMR magnitude in the neurofeedback training group posttreatment. Results for drug use, mean theta magnitude, and number of commission errors posttreatment were not significant.

Pearson correlations revealed no significant correlations (*P*>.05) between the difference in SMR or theta magnitude by the end of the training and behavioral outcome measures.

**Table 1:** Baseline sample characteristics (N=42).

Sample characteristics	Neurofeedback training group	Treatment-as-usual only group
Age (years), mean (range)	38.00 (21-55)	38.57 (26-55)
Months in treatment at T0, mean (range)	91.90 (19-248)	95.30 (10-290)
Axis I and II diagnoses, mean (range)	4.5 (2-8)	4.6 (1-7)
Psychopathy score (PCL-R^a^), mean (range)	23.86 (15-32)	23.77 (15-36)

^a^PCL-R: Psychopathy Checklist–Revised.

**Table 2:** Type of substance use diagnosis for the neurofeedback training group and the treatment-as-usual only group (N=42).

Type of substance use diagnosis	Neurofeedback training group, n (%)	Treatment-as-usual only group, n (%)
Alcohol	9 (42.86)	13 (61.90)
Cannabis	12 (61.90)	19 (90.48)
Amphetamines	4 (19.05)	8 (38.10)
Opioids	1 (4.76)	4 (19.05)
Cocaine	5 (23. 81)	9 (42.86)
Sedative	2 (9.52)	1 (4.76)
Other	3 (14.29)	0 (0)

**Table 3:** Comorbid axis I and II diagnosis and index offense for the neurofeedback training group and the treatment-as-usual (TAU) only group (N=42).

Comorbid axis I and II diagnosis and index offense		Neurofeedback training group, n (%)	Treatment-as-usual group, n (%)
**Comorbid axis I disorder**			
	Pervasive developmental disorder^a^	2 (9.52)	0 (0)
	ADHD^b^	6 (28.57)	3 (14.29)
	Disorders in the schizophrenia spectrum	12 (57.14)	10 (47.62)
	Mood and anxiety disorder	2 (9.52)	2 (9.52)
	Pedophilia	1 (4.76)	1 (4.76)
	PTSD^c^	2 (9.52)	3 (14.29)
**Comorbid axis II disorder**			
	Antisocial personality disorder	8 (38.10)	7 (33.33)
	Borderline personality disorder	2 (9.52)	4 (19.05)
	Personality disorder not otherwise specified	7 (33.33)	7 (33.33)
	Avoidant personality disorder	1 (4.76)	1 (4.76)
**Index offense^d^**			
	Homicide	9 (42.86)	7 (33.33)
	Sexual offense	2 (9.52)	4 (19.05)
	Arson	1 (4.76)	2 (9.52)
	Violence	3 (14.29)	3 (14.29)
	Threat against life	4 (19.05)	3 (14.29)
	Theft	2 (9.52)	2 (9.52)

^a^Pervasive developmental disorder: Autism spectrum disorder, Asperger disorder, developmental disorder not otherwise specified.

^b^ADHD: all types of attention-deficit disorder.

^c^PTSD: posttraumatic stress disorder.

^d^Index offense: in case of more than one index offense, the most serious one is reported, based on the classification given in the study by Nieuwenhuizen et al [3].

**Table 4:** Main outcome measures of repeated measures analysis. Sample sizes were N=42, except for scores on drug use which was n=40. Significant results are in italics.

Outcome measure	Neurofeedback training, mean (SD)		Treatment-as-usual, mean (SD)		Post- or premeasurement						
	T0	T1	T0	T1	Time		Group		Time x Group		η^2^
					*F*	*P* value	*F*	*P* value	*F*	*P* value	
BIS-11^a^	67.05 (11.05)	63.10 (10.88)	63.33 (12.23)	62.62 (11.5)	3.02	.09	.41	.52	1.45	.24	.03
BIS-11 motor	24.10 (6.24)	21.67 (3.97)	21.81 (4.49)	20.95 (4.42)	5.61	*.02*	1.27	.27	1.28	.26	.03
BIS-11 nonplanning	25.86 (4.21)	25.05 (.5.82)	25.3 (6.18)	25.6 (6.03)	.14	.71	.00	.99	.60	.44	.02
BIS-11 attentional	17.10 (3.36)	16.38 (3.2)	16.19 (4.06)	16.05 (3.79)	.96	.33	.36	.55	.43	.52	.01
DAQ-SF^b^	44.19 (17.77)	36.38 (20.45)	42.72 (17.48)	39.24 (16.49)	6.23	*.02*	.02	.89	.92	.34	.02
Commission errors	2.05 (3.44)	1.52 (1.91)	1.00 (1.00)	1.14 (1.42)	.30	.59	1.59	.21	.92	.34	.02
Drug use	.53 (.64)	.38 (.50)	.23 (.31)	.22 (.35)	1.67	.20	2.91	.10	1.27	.27	.03
Theta	3.94 (3.67)	4.31 (3.53)	2.54 (1.64)	2.75 (1.72)	1.81	.19	3.12	.09	.14	.71	.00
SMR^c^	1.01 (.52)	1.23 (.66)	.65 (.39)	.64 (.35)	5.00	*.03*	10.56	*.00*	5.47	*.02*	.12

^a^BIS-11: Barratt Impulsivity Scale-11.

^b^DAQ-SF: Desire for Alcohol Questionnaire.

^c^SMR: sensorimotor rhythm.

## Discussion

### Principal Findings

This RCT was conducted to investigate to what extent a theta/SMR neurofeedback training protocol in combination with TAU is able to reduce impulsivity and symptoms of SUD in a population of male forensic psychiatric patients residing in an FPC. The RCT compared a neurofeedback training group of 21 patients who received neurofeedback training in addition to TAU with a control group of 21 patients receiving TAU only. Changes in targeted frequency bands and changes in levels of impulsivity, drug craving, and drug intake posttreatment were examined in patients in the neurofeedback training group and compared with patients in the TAU only group. Results indicate that SMR magnitude showed a significant increase posttreatment in the neurofeedback training group, whereas theta magnitude did not show any changes. Surprisingly, patients in the neurofeedback training group had significantly higher SMR magnitude pretreatment than patients in the TAU only group.

Levels of drug craving and motor impulsivity as assessed with the BIS-11 decreased equally for patients in the neurofeedback training group and the TAU only group. Therefore, the combination of TAU and neurofeedback training was not more effective than TAU only. Other measures of impulsivity and number of drug use did not change posttreatment.

To the best of our knowledge, this is the first RCT study investigating the effects of neurofeedback training in a population of forensic psychiatric patients. Studies on investigating neurofeedback training have steadily increased in the past two decades, but neurofeedback training is rarely used as a treatment option for forensic psychiatric patients. This could partially be due to the fact that these patients usually present with a variety of disorders, and research on the effects of neurofeedback usually exclude patients with comorbid disorders [39-42]. Furthermore, practitioners might be hesitant to employ a treatment modality for which the efficacy in such a complex patient population is yet to be demonstrated.

The fact that effects of neurofeedback training were not superior to TAU only has also been observed in other studies that applied neurofeedback in an attempt to reduce levels of impulsivity. Bink et al [43] employed a theta/SMR protocol in children with ADHD but found the combination of TAU and neurofeedback as effective as TAU only. Schönenberg et al [44] also found no superiority of a theta/beta neurofeedback training over a meta-cognitive therapy or even a sham neurofeedback condition. Both Bink et al [43] and Schönenberg et al [44] applied the training in subjects with a single, well-defined disorder without any comorbidities. Hence, it can be argued that for patients with multiple disorders and characterized by high levels of impulsivity, finding behavioral improvements due to neurofeedback training may be even more difficult.

The results of this study also raise the question as to how participants’ failure to decrease theta activity over the course of the training is associated with the lack of behavioral improvements posttreatment. To date, there are no clear guidelines about how many neurofeedback training sessions are actually needed to achieve significant treatment effects; it is possible that improvements in the theta frequency band could have been achieved with more sessions. Bink et al [43] found that adolescents with ADHD were better able to suppress theta frequency by the end of the training sessions than at the beginning of neurofeedback training. In the study by Bink et al [43], 37 sessions were employed, but they still did not observe an effect of the neurofeedback training in the reduction of impulsivity. It may be the case that the 20 sessions of neurofeedback provided in this study simply were not enough for this patient group to learn to regulate the theta frequency band. However, patients’ inability to adhere to the training schedule of 2 neurofeedback sessions a week might be indicative of the feasibility of a neurofeedback protocol that employs even more sessions. Throughout this study, it was difficult to keep patients engaged in the study. Although the specific patient population at hand is difficult to engage in treatment no matter which treatment is applied, the fact that none of the patients in the neurofeedback training group were able to attend 2 sessions a week is concerning. This was partially due the fact that neurofeedback software is still in its infancy and options concerning the employed training methods are limited. In most cases, the implemented video games are quite simplistic. A lot of patients reported that they found the intervention dull, which most likely was of negative influence on treatment motivation. An abbreviated protocol might be better suited for this patient population in terms of keeping patients engaged in the training. In addition, as results of this study showed no significant relation between patient’s reduction in theta magnitude and behavioral outcome measures, it remains unclear as to whether (more) improvements in theta activity regulation can lead to (better) clinical improvements at the behavioral level. However, patients did manage to increase SMR magnitude posttreatment. It is possible that the SMR frequency band is easier to regulate with neurofeedback training. In a recent study by Fielenbach et al [45], which focused on whether forensic psychiatric patients are actually able to learn to regulate cortical activity through neurofeedback training, more patients were able to systematically increase SMR activity as opposed to reducing theta activity. In a study by Doppelmayr and Weber [46], healthy participants were better able to regulate SMR activity than to change the theta/beta ratio, and a recent study by Janssen et al [39] showed that adolescents were not able to inhibit their theta frequency but did manage to increase beta activity.

It is unclear why patients in the neurofeedback training group showed higher pretreatment SMR magnitude than patients in the TAU only group, as patients’ distribution over groups was random. However, previous studies with healthy participants have suggested that pretreatment SMR magnitude is a predictor of participants ability to increase of SMR magnitude over the course of neurofeedback training [47-50]. Possibly, the higher pretreatment levels of SMR magnitude contributed to the finding that patients did manage to increase SMR magnitude over the course of training.

Recently, quantitative electroencephalography (QEEG)-guided neurofeedback protocols are increasingly implemented in clinical practice. With these protocols, pretreatment EEG deviations are first assessed and the applied neurofeedback protocol then focusses on treating these EEG deviations, as opposed to applying a standard neurofeedback protocol to all participants. Although there is also discussion in the literature about the use of QEEG approach of neurofeedback treatment (eg, Johnson and Bodenhamer-Davis [51]), this approach fits with the rise of personalized medicine in the past decade, where a treatment approach tailored to the individual is applied rather than a one-size-fits-all approach. Especially for forensic psychiatric patients, usually presenting with a wide range of comorbidities, manifesting through various deviations in EEG-frequencies, this might be a more suitable approach than applying standardized neurofeedback protocols.

### Limitations and Recommendations for Future Studies

Patients taking prescription medication were allowed to keep taking these medications during the course of the study. Given the special setting where this study was conducted, limiting medication intake would have severely hampered patient recruitment. However, almost all types of medication commonly prescribed for forensic psychiatric patients tend to have effects on EEG frequencies. Several studies have shown that stimulant medication such as methylphenidate normalizes EEG frequencies and may lead to a reduction of theta band magnitude and an increase in low beta bands magnitude [49,50]. Medication for disorders in the schizophrenia spectrum, such as clozapine, have been shown to increase theta activity (Hyun et al [52]). It is very well possible that the results of this study were, to some extent, influenced by the type and/or dosage of patients’ medication. A theta/SMR neurofeedback protocol might not lead to significant changes in EEG-frequencies when these frequency bands are already normalized due to use of medication, although this remains speculative. In this study, changes in medication were insufficiently tracked during the course of the study. Future studies should investigate the effects of medication on the EEG spectrum more closely before applying neurofeedback or should at least control for medication intake during the analysis to achieve more conclusive results. Another limitation concerning medication is the fact that some medication, such as aripiprazole, is known to have positive effects on levels of craving (Beresford et al [53]), which could have influenced the results on the craving questionnaire DAQ-SF.

Moreover, with the patient sample of this study, there was heterogeneity concerning substances used by study participants. This is quite common in patients with SUD, as many patients are polydrug abusers. This may have altered the results and potentially influenced the effects of neurofeedback in these patients. In addition, we followed treatment facility policy, where a refusal to undergo drug testing is scored as having a positive drug testing. There is no way to be certain that patients who refused to undergo drug testing did, in fact, use illicit substances. However, given our clinical experience, patients refusal to undergo drug testing usually lies in the fact that they have relapsed in drug use, as patients have no reason to refuse to undergo drug testing other than fear of having drug use exposed. Refusal to undergo drug testing will result in the loss of privileges, so that refusing to undergo drug testing comes at a reasonable cost to patients.

In addition, the fact that none of the patients in the neurofeedback training group were able to complete the training in the scheduled amount of time could have influenced the results. Possibly, results achieved in terms of enhancing or inhibiting EEG frequencies were lost in between sessions because patients were not able to follow the scheduled training sessions. To date, there is no conclusive research indicating the ideal number of neurofeedback training sessions or the most beneficial interval time in between training sessions. For this study, adhering to a very strict training schedule, where patients would have been excluded from further participation whenever they missed a session, would have resulted in a very high number of dropouts and consequently in lower power of the results found. Nonetheless, the failure of patients’ adherence to the schedule could have been of influence on the study results. Another limitation of this study is that a sham-neurofeedback control group was not added to the study. Although some authors challenge the use of a sham neurofeedback condition [54], as even a sham-based neurofeedback training can lead to treatment outcomes, it could have been useful to add a waiting list group as an untreated control condition.

Future studies should also investigate whether results in terms of patients’ ability to increase or decrease their frequency magnitude vary when manually adjusted thresholds are applied versus when automatically adjusted thresholds are applied. Manually adjusted thresholds are subject to the expertise of the neurofeedback trainer; therefore, they are also subject to, for example, inattention of the trainer. Automatically adjusted thresholds provide a more objective way of adjusting thresholds, which might be better suitable for scientific purposes.

### Conclusions

This study highlights that more research is needed to assess the efficacy of a theta/SMR neurofeedback protocol for the reduction of impulsivity, drug craving, and drug intake in forensic psychiatric patients with substance abuse problems. Results showed that patients were unable to learn the whole neurofeedback protocol as they did not succeed in reducing theta activity. Future research should focus on assessing which patients will be able to benefit from neurofeedback training at an early stage of the employed training sessions. Given that neurofeedback training is often times applied in vulnerable patient populations such as children, adolescents, and patients with severe mental illness or addiction, it can be considered unethical to enroll these patients in any treatment with the knowledge that it will most likely not lead to beneficial outcomes in terms of reduction of clinical symptoms. Weber et al [55] have made an important start with their research on predicting successful learning of SMR neurofeedback in healthy participants. This research needs to be extended to clinical populations.

## Appendix

Multimedia Appendix 1 CONSORT‐EHEALTH checklist (V 1.6.1).

## Abbreviations

ADHD: attention-deficit hyperactivity disorder
BIS-11: Barratt Impulsivity Scale-11
DAQ-SF: Desire for Alcohol Questionnaire
EEG: electroencephalographic
FPC: forensic psychiatric center
RCT: randomized controlled trial
SMR: sensorimotor rhythm
SUD: substance use disorders
TAU: treatment-as-usual
